# Gene stacking of multiple traits for high yield of fermentable sugars in plant biomass

**DOI:** 10.1186/s13068-017-1007-6

**Published:** 2018-01-09

**Authors:** Aude Aznar, Camille Chalvin, Patrick M. Shih, Michael Maimann, Berit Ebert, Devon S. Birdseye, Dominique Loqué, Henrik V. Scheller

**Affiliations:** 1 Feedstocks Division, Joint BioEnergy Institute, Lawrence Berkeley National Laboratory, Berkeley, CA 94720 USA; 2 Ecole Normale Supérieure de Cachan, 94230 Cachan, France; 3 Technical University of Denmark, 2800 Kgs. Lyngby, Denmark; 4 Department of Plant and Environmental Sciences, University of Copenhagen, 1871 Frederiksberg, Denmark; 5 School of BioSciences, University of Melbourne, Melbourne, VIC 3010 Australia; 6 INSA de Lyon, CNRS, UMR5240, Microbiologie, Adaptation et Pathogénie, Université Claude Bernard Lyon 1, 69622 Villeurbanne, France; 7 Department of Plant and Microbial Biology, University of California Berkeley, Berkeley, CA 94720 USA

**Keywords:** Plant cell wall, Galactan, Arabidopsis, Pectin, jStack, Xylan, Lignin, Plant engineering

## Abstract

**Background:**

Second-generation biofuels produced from biomass can help to decrease dependency on fossil fuels, bringing about many economic and environmental benefits. To make biomass more suitable for biorefinery use, we need a better understanding of plant cell wall biosynthesis. Increasing the ratio of C6 to C5 sugars in the cell wall and decreasing the lignin content are two important targets in engineering of plants that are more suitable for downstream processing for second-generation biofuel production.

**Results:**

We have studied the basic mechanisms of cell wall biosynthesis and identified genes involved in biosynthesis of pectic galactan, including the GALS1 galactan synthase and the UDP-galactose/UDP-rhamnose transporter URGT1. We have engineered plants with a more suitable biomass composition by applying these findings, in conjunction with synthetic biology and gene stacking tools. Plants were engineered to have up to fourfold more pectic galactan in stems by overexpressing GALS1, URGT1, and UGE2, a UDP-glucose epimerase. Furthermore, the increased galactan trait was engineered into plants that were already engineered to have low xylan content by restricting xylan biosynthesis to vessels where this polysaccharide is essential. Finally, the high galactan and low xylan traits were stacked with the low lignin trait obtained by expressing the *QsuB* gene encoding dehydroshikimate dehydratase in lignifying cells.

**Conclusion:**

The results show that approaches to increasing C6 sugar content, decreasing xylan, and reducing lignin content can be combined in an additive manner. Thus, the engineered lines obtained by this trait-stacking approach have substantially improved properties from the perspective of biofuel production, and they do not show any obvious negative growth effects. The approach used in this study can be readily transferred to bioenergy crop plants.

**Electronic supplementary material:**

The online version of this article (10.1186/s13068-017-1007-6) contains supplementary material, which is available to authorized users.

## Background

By taking advantage of the massive energetic potential of plant lignocellulosic biomass, second-generation biofuels can be produced from a broad spectrum of renewable carbon sources without creating direct competition with the food production market. Lignocellulosic biomass is largely composed of secondary plant cell walls, which are thick walls surrounding some differentiated cells such as xylem vessels. Secondary walls are mainly composed of cellulose, hemicelluloses, and lignin [1], and constitute the most abundant biomaterials on Earth. Cell wall polysaccharides can be enzymatically degraded to release monosaccharides through a process known as saccharification. Subsequently, microbes can be used to convert monosaccharides to produce bioethanol or other bioproducts. However, several characteristics of lignocellulosic biomass limit the yield of this process, making it expensive [2].

Secondary cell wall polysaccharides are embedded in lignin, a polymer of cross-linked aromatic alcohols that protect the cell wall polysaccharides from enzymatic degradation by plant pathogens and pests, e.g., in case of nematode infection [3], and contribute to biomass recalcitrance. Up to 35% of secondary cell walls are hemicelluloses, which in angiosperms are composed mainly of pentoses such as xylose and arabinose [4]. Because pentoses are less fermentable by microorganisms than hexoses [5, 6], increasing the hexose/pentose ratio is one approach to improving biomass for biofuel production. Due to the low density of the lignocellulosic biomass, transportation costs to processing facilities are another limiting factor, especially for biomass from grasses [7]. Engineering of plants with modified secondary cell walls more suitable for downstream processing could reduce costs and facilitate biofuel production from lignocellulosic biomass.

Nevertheless, modifying the cell wall also remains challenging because of its central role in many plant functions. Different groups have already investigated several approaches to making lignocellulosic biomass more suitable for conversion into bioethanol [8]. Genetic engineering efforts to reduce lignin content typically employ techniques to constitutively repress lignin biosynthesis, e.g., by RNAi. However, these strategies frequently result in reduced plant size, and often more than one copy of the biosynthetic gene must be targeted to achieve reduced lignin [9–11]. Recently, we developed a gain-of-function strategy allowing a decrease in lignin content without any visible effect on plant growth [12]. The *Arabidopsis thaliana CINNAMATE*-*4*-*HYDROXYLASE* (*C4H*) promoter was used to express the *Quinate and Shikimate Utilization B* (*QsuB*) gene encoding the 3-dehydroshikimate dehydratase from *Corynebacterium glutamicum* in lignified tissues of *A. thaliana* [12]. By converting 3-dehydroshikimate into protocatechuic acid, the QsuB enzyme produces two effects: (1) it limits the availability of shikimate, a precursor for lignin biosynthesis and a cofactor of hydroxycinnamoyl transferase and (2) it produces an inhibitor of the same transferase [13, 14]. Biomass from plants expressing *QsuB* in lignified tissues exhibits a 50% decrease in lignin content and shows improved saccharification efficiency.

One approach to increase the hexose/pentose ratio in lignocellulosic biomass is to increase the proportion of hexose-rich polysaccharides in secondary cell walls. β-1,4-Galactan is entirely composed of galactose residues and is found as sidechains of rhamnogalacturonan I in pectin of primary cell walls [15]. Pectin, including β-1,4-galactan, is not abundant in secondary cell walls except in gelatinous fibers. Gelatinous fibers are found in plants such as flax and in tension wood, a specific type of wood that plays a role in maintaining appropriate plant growth under mechanical stress [16]. Tension wood of aspen trees has been reported to contain 10% of β-1,4-galactan, which is hypothesized to induce gel-like properties, conferring the contractile driving force of tension wood [16]. Our previous work showed that the glycosyltransferase Galactan Synthase 1 (GALS1) is a β-1,4-galactan synthase involved in the biosynthesis of pectic galactan in the Golgi apparatus [17]. Constitutive overexpression of *GALS1* in Arabidopsis increased the amount of galactose in leaf cell walls by 50% without an apparent effect on plant growth.

Moreover, the co-overexpression of *GALS1* and the cytosolic *UDP*-*glucose/UDP*-*galactose*-*4*-*epimerase 2* (*UGE2*) [18] under the control of the 35S promoter led to an 80% increase in galactose in stem cell walls [19]. As UDP-galactose residues are polymerized in the Golgi apparatus, whereas UDP-galactose is synthesized in the cytosol [15], UDP-galactose transport from cytosol to Golgi could become limiting when *GALS1* and *UGE2* are both overexpressed. Recently, the Golgi-localized UDP-Rhamnose/UDP-Galactose Transporter 1 (URGT1) was shown to be involved in transport of UDP-galactose into the Golgi apparatus [20]. Overexpression of URGT1 led to increased β-1,4-galactan accumulation in Arabidopsis leaves, indicating that UDP-galactose transport may indeed be limiting for galactan biosynthesis. In this study, we aimed to overcome the limitation in UDP-galactose in the Golgi lumen by co-overexpressing *URGT1* with *GALS1* and *UGE2*.

Another approach to increase the hexose/pentose ratio in lignocellulosic biomass is to decrease the proportion of pentose-rich polysaccharides. The glycosyltransferase Irregular Xylem 7 (IRX7) is involved in the biosynthesis of xylan, a polymer of β-1,4-linked xylose units, which are highly abundant in secondary cell walls [21]. Compared to wild-type plants, *irx7* loss-of-function mutants have a lower xylan content and exhibit severe dwarfism due to collapsed xylem vessels and the consequent impairment of water and nutrient transport [21]. Using the *VND7* vessel-specific promoter to express the *IRX7* coding sequence in the *irx7* mutant, the growth phenotype is rescued while the content of xylose residues in stem cell walls is still reduced by 20% as compared to non-engineered lines [22]. Here, we utilized this genetically engineered low pentose background and engineered increased galactan content to further increase the C6 to C5 ratio.

The NAC (NAM/ATAF1/CUC2) Secondary cell wall Thickening-promoting factor 1 (NST1) transcription factor is a master regulator controlling secondary cell wall biosynthesis in fiber cells, which provide mechanical support to vessels in stems [23]. *NST1* is a potential target for the modification of cell wall thickness. However, its constitutive overexpression leads to the formation of ectopic secondary cell wall thickening and inhibits plant growth [23]. A system allowing overexpression of *NST1* without any negative effect on plant growth has been developed by Yang et al. [24]. A downstream promoter induced by NST1 is used to express a new chimeric copy of *NST1*, creating an artificial-positive feedback loop (APFL) and leading to the over-accumulation of NST1 in fiber cells only. Plants expressing a chimeric copy of *NST1* under the control of one of its target promoters have fiber cells with thicker secondary cell walls and, accordingly, lignocellulosic biomass obtained from their stems is of a higher density [19, 23].

In the present work, genetic engineering was used to modify cell wall composition and thickness in Arabidopsis, with the aim of increasing the C6 to C5 ratio, reducing biomass recalcitrance, and increasing the lignocellulosic biomass density obtained from plant stems for biofuel production.

Overexpression of *URGT1* together with *GALS1* and *UGE2* increased the cell wall galactan content in stems without causing any growth defects. The use of constitutive promoters was compared with the use of secondary cell wall-specific promoters, which limit transgene expression to lignified tissues that constitute the major part of lignocellulosic biomass.

We also stacked several previously described cell wall engineering strategies to evaluate additive effects and compatibility. First, increased galactan content and decreased lignin content traits were combined by overexpressing *QSUB* together with *GALS1*, *UGE2,* and *URGT1*. Secondly, increased cell wall thickness was combined with higher galactan content and/or lower lignin content through the overexpression of NST1, creating an APFL that increased secondary wall density in interfascicular fiber cells. Third, all these modifications have been combined with a decrease in xylan content by overexpressing the gene sets described above in a vessel-complemented xylan-deficient background [22].

## Results

### Design, construction, and stacking of multiple genes for improved biomass traits

**Fig. 1:**
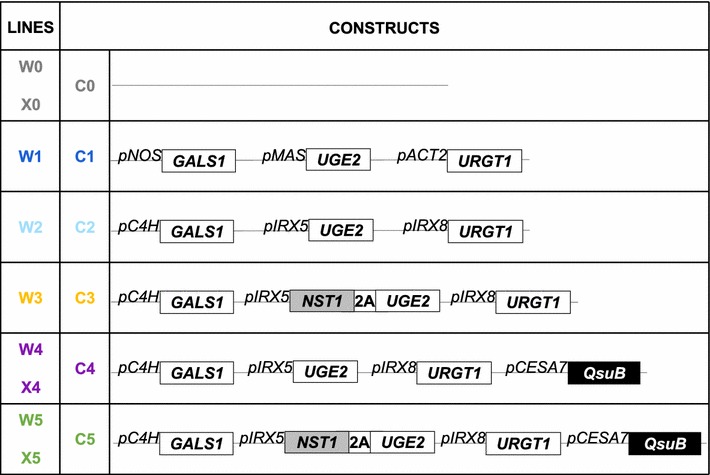
jStack constructs for the generation of multiple traits engineered Arabidopsis. Lines W0, W1, W2, W3, W4 and W5 express constructs C0, C1, C2, C3, C4 and C5, respectively, in Col-0 WT background. Lines X0 and X4 express constructs C0 and C4, respectively, in *irx7/irx7* pVND7:IRX7 xylan-engineered (XE) background. White squares represent galactan biosynthesis-related ORF, gray square represents secondary cell wall artificial-positive feedback loop-related ORF, and black square represents decreasing lignin-related ORF. *pNOS*, *pMAS* and *pACT2* are constitutive promoters, and *pC4H*, *pIRX5*, *pIRX8* and *pCESA7* are promoters specifically expressed in secondary cell wall producing cells. The foot-and-mouth disease virus 2A sequence ensures NST1 and UGE2 protein coordinated expression

We aimed to overexpress *GALS1*, *UGE2,* and *URGT1* involved in galactan biosynthesis; the *NST1* coding sequence to create an APFL increasing cell wall thickness in fibers; and the *QsuB* gene responsible for lignin decrease. In this work, the jStack method was used to clone all the transgenes in a single binary vector, allowing a single step transformation of Arabidopsis plants and resulting in the co-segregation of the different transgenes [25]. Five constructs were designed with the combinations of transgenes depicted in Fig. 1.

The constructs named C1 and C2 were designed to improve galactan engineering in Arabidopsis (Fig. 1). In C1, the expression of *GALS1*, *UGE2* and *URGT1* coding sequences is under the control of *pNOS (Nopaline Synthase)*, *pMAS* (*Mannopine Synthase*) and *pACT2* (*Actin 2*) constitutive promoters [25], respectively. In C2, the expression of *GALS1*, *UGE2,* and *URGT1* coding sequences is under the control of the secondary cell wall-specific *pC4H*, *pIRX5,* and *pIRX8* promoters, respectively. In Arabidopsis, *IRREGULAR XYLEM 5* (*IRX5*), also named *CESA4*, encodes one of the catalytic subunits of the cellulose synthase complex [26] and *IRREGULAR XYLEM 8* (*IRX8*), also named *GAUT12*, encodes a protein with putative galacturonosyltransferase activity that is required for xylan biosynthesis [27].

The construct C3 was designed to increase cell wall thickness specifically in fibers by including the NST1 APFL along with transgenes from the C2 construct (Fig. 1). The coding sequence of *NST1* was fused with the *UGE2* coding sequence using a 2A-peptide as described previously [19]. The 2A-peptide allows coordination of the expression of multiple proteins and polyprotein cleavage in plants [28]. The *NST1*-*2A*-*UGE2* synthetic sequence is driven by the *pIRX5* promoter.

Because we also aimed to reduce the amount of lignin in fibers of the galactan-engineered lines, the *QsuB* coding sequence [12] was stacked with C2 construct synthetic genes, resulting in the generation of construct C4, or with C3 construct synthetic genes, resulting in the generation of construct C5 (Fig. 1) In both C4 and C5, the expression of the bacterial 3-dehydroshikimate dehydratase encoded by the *QsuB* sequence is controlled by the promoter of *CESA7*, which is one of the catalytic subunits of cellulose synthase in secondary walls [26].

Wild-type Arabidopsis plants were independently transformed with constructs C1, C2, C3, C4, C5, or the C0 empty vector control construct. The corresponding T3 generation plants from these lines will be, respectively, named W1, W2, W3, W4, W5, or W0. To further increase the C6:C5 ratio in cell walls, the xylan-engineered line *irx7/irx7 pVND7:IRX7* was used as genetic background [22]. The *irx7/irx7 pVND7:IRX7* line, named here xylan-engineered (XE) line, was independently transformed with constructs C0, C4 or C5. Plants from these lines were, respectively, named X0, X4, or X5.

### Morphological phenotypes of the engineered lines

**Fig. 2:**
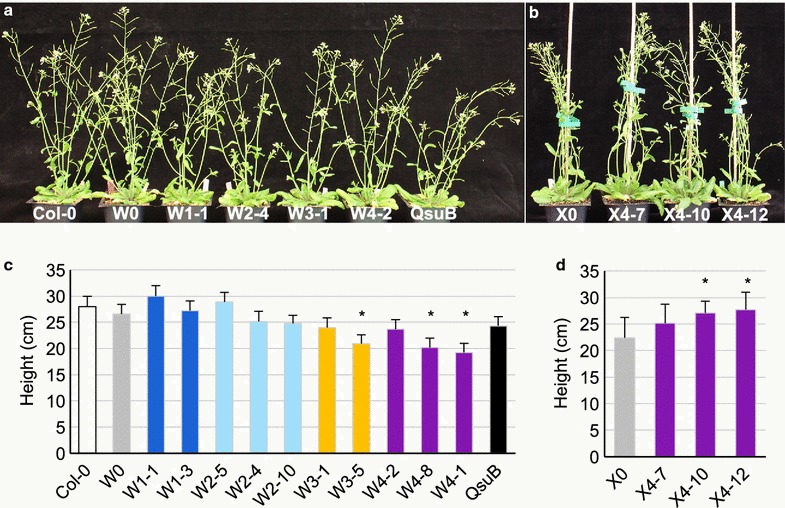
Morphological phenotypes of engineered lines. Pictures (**a**, **b**) and main stem height average (**c**, **d**) of seven-week-old engineered plants in Col-0 wild type (**a**, **c**) and *irx7/irx7 pVND7:IRX7* xylan-engineered (**b**, **d**) backgrounds. Bars: SD, *n* = 6. *p < 0.05 with Student’s *t* test, in comparison to W0 (**c**) or X0 (**d**)

In general, no obvious differences were observed in inflorescence stem morphology and growth of W- and X-engineered lines in comparison to Col-0, W0 and X0 controls (Fig. 2), except for some of the W3 and W4 lines, where the inflorescence stems were shorter than those of the controls (Fig. 2c). Interestingly, two of the three X4 lines analyzed were taller at maturity than the X0 line (Fig. 2d). Inflorescence stems of plants from the W5 and X5 lines were not able to stand fully upright (see Additional file 1). Because of the incompatibility of this phenotype with our engineering strategy, W5 and X5 plants were not included in our further studies.

The following analysis focuses on two or three independent engineered lines for each construct: two independent lines for W1 (W1-1, W1-3), W3 (W3-1, W3-5) and three independent lines for W2 (W2-5, W2-4, W2-10), W4 (W4-2 W4-8, W4-1) and X4 (X4-7, X4-10, X4-12).

### Expression levels of stacked genes in engineered lines

**Fig. 3:**
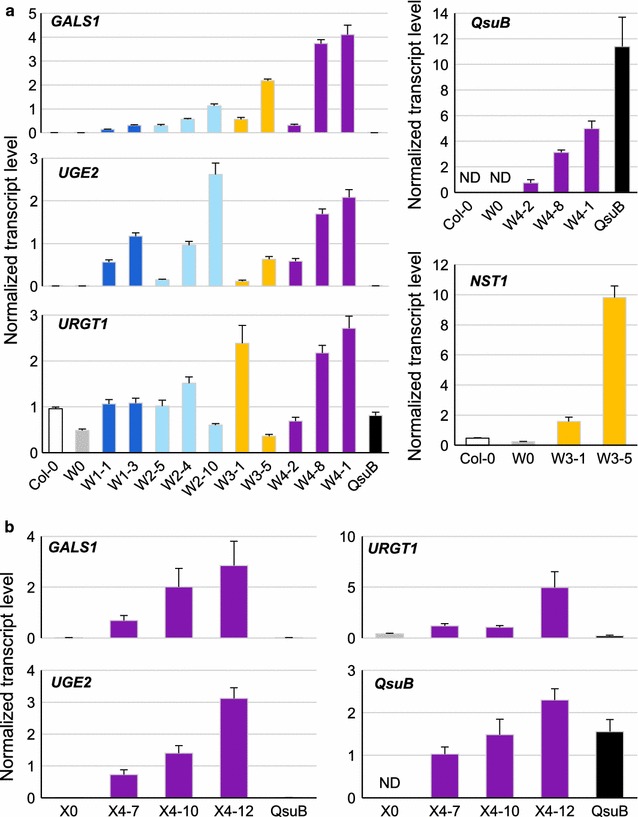
Stacked genes expression level in stems of engineered lines. The expression of indicated genes was monitored by qPCR using cDNA from inflorescence stems tissues of W- (**a**) and X-(**b**) engineered lines. Gene expression was normalized against the geometric average of transcript levels of three constitutively expressed genes (*UBQ10*, *PP2AA3* and *MON1*). Bars: SD of the normalized ratio, *n* = 3. ND, not detected

The accumulation of *GALS1*, *UGE2*, *URGT1*, *NST1,* and *QsuB* transcripts was determined by qPCR (quantitative real-time polymerase chain reaction) in inflorescence stem tissues of W- and X-engineered lines (Fig. 3). In general, there was a correlation in expression between the stacked genes in the individual lines, as might be expected since they will be affected. However, independent lines resulting from transformations with the same construct showed large differences in transcript levels of the introduced genes. Compared to controls, transcripts of *GALS1* and *UGE2* accumulated 43- to 818-fold in W1 and W2 lines (Fig. 3a). *URGT1* transcript levels did not increase substantially in the W1 and W2 lines, but increased three- to 11-fold in several of the W3, W4, and X4 lines. In the W3, W4 and X4 lines, the transcripts of *GALS1* and *UGE2* were generally increased even more than in the W1 and W2 lines. In W3-1 and W3-5 lines, *NST1* transcripts accumulated, respectively, five- and 42-fold compared to controls. The *QsuB* transcripts were only detected in stem tissues of W4 and X4 lines, as expected, and levels varied between independent lines (Fig. 3a, b). *QsuB* transcript accumulations were lower in the W4 and X4 lines than in the *QsuB* control in which *QsuB* is under the control of a stronger pC4H promoter. In conclusion, healthy T3 plants expressing galactan galactan-engineered genes and NST1 APFL or *QsuB* were successfully generated for further characterization.

### Immunodetection of galactans in stems of engineered lines

**Fig. 4:**
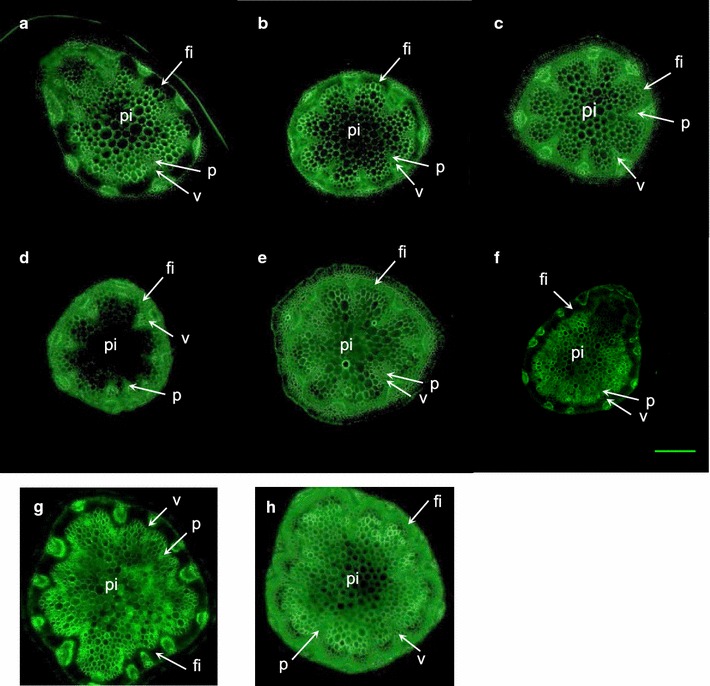
Galactan detection in stem cross-sections of engineered lines. Cross-section of seven-week-old stem samples were labeled with LM5 (anti-β-1,4-galactan) antibody and binding was revealed with an anti-rat IgG antibody coupled with FITC. **a** W0; **b** W1-1; **c** W2-5; **d** W3-1, **e** W4-8, **f** pC4H::QsuB; **g** X0, **h** X4-12. Scale bar = 20 µm. The general location of pith (pi), vessels (v), phloem (p), and interfascicular fibers (fi) is indicated

To confirm that expression of genes controlling galactan biosynthesis in fibers resulted in targeted galactan enrichment, stem cross-sections from engineered plants were analyzed by immunodetection of galactan using the β-1,4-galactan-specific LM5 antibody (Fig. 4). While galactan was mainly detected in the cortex, phloem, and pith cell walls of W0 and X0 stems (Fig. 4a, g), it was also present in fiber cell walls of all W- and X-engineered lines. The LM5 signal in fibers was weakly detected in W1-1 and W2-5 stems (Fig. 4b, c). In a W3-1 stem cross-section, no LM5 fluorescence was detected in the pith, but a bright signal emanated from the walls of phloem and fiber cells. In W4-8 stems, LM5 signal was detected in pith, phloem, and fiber cell walls (Fig. 4d, e). While there was no signal in X0 fibers (Fig. 4g), LM5-associated fluorescence was detected in fibers of X4-12 (Fig. 4h).

### Cell wall monosaccharide composition of engineered lines

**Fig. 5:**
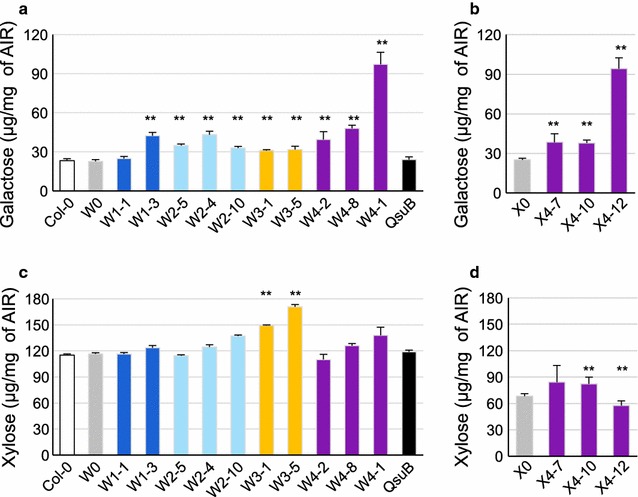
Galactose and xylose content in stem cell walls of engineered lines. Galactose (**a**, **b**) and xylose (**c**, **d**) content from alcohol insoluble residue (AIR) of stem cell walls of W- (**a**, **c**) and X-(**b**, **d**) engineered lines, hydrolyzed with TFA acid, and analyzed by HPAEC-PAD. Sugar residues not show here are displayed in Additional file 2. Error bars: SD, *n* = 3. **p < 0.01 with Student’s *t* test, in comparison to W0 (**a**, **c**) or X0 (**b**, **d**)

After TFA (triflouroacetic acid) hydrolysis, the monosaccharide content of dry stems from engineered plant lines was analyzed by High-Performance Anion Exchange Chromatography with Pulsed Amperometric Detection (HPAEC-PAD). Figure 5 highlights galactose and xylose, while Additional file 2 shows all sugar residues measured. As expected, in the engineered lines in wild-type background, galactose content in the stem was significantly modified compared to Col-0, W0, and pC4H::QsuB controls (Fig. 5a). A 50% increase in galactose content was detected in W1-8, W2-4, W2-5, W2-10, and W4-2 stems, and up to two- to fourfold increase in W4-8 and W4-1, respectively (Fig. 5a). X4 lines also contained more galactose than X0 without affecting xylose content to a large extent. X4-7 and X4-10 contained 30% more galactose and X4-12 contained almost three times more galactose residues than X0 stems (Fig. 5b). In conclusion, when galactan biosynthesis is boosted in fibers, the galactose content is increased in biomass from stems.

### Biomass accumulation in inflorescence stems

**Fig. 6:**
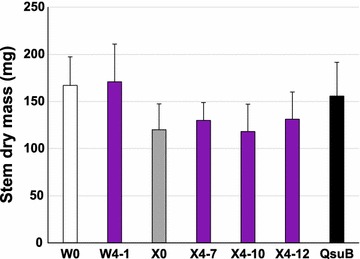
Biomass yield of inflorescence stems. Plants were grown until maturity and the mass of the senesced, dry stems determined. The data are averages ± SE for four separate experiments, each with three plants per genotype. No significant differences were found (ANOVA, *p* > 0.3)

To investigate if the large increase in C6 to C5 ratio in the engineered W4-1 line and the X4 lines was associated with a change in overall biomass yield, we determined the mass of senesced stems (Fig. 6). None of the differences were significant (ANOVA, *p* > 0.3), and this was also true when the X4 lines were analyzed together (ANOVA and Tukey’s test, *p* > 0.1 for X4 lines being different from any other lines).

### Modification of mechanical properties of engineered plant inflorescence stems

**Fig. 7:**
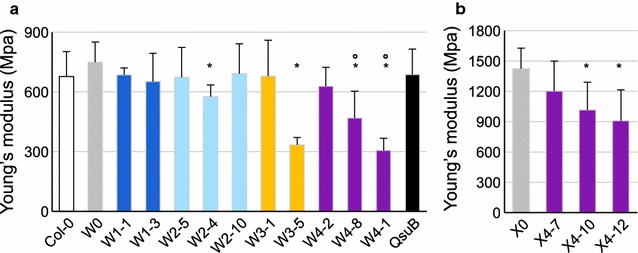
Young’s modulus measurements of inflorescence stems. The three-point bending force of the main inflorescence stem of seven-week-old W- (**a**) or X-(**b**) engineered lines was tested. Error bars: SD, *n* = 6. *p < 0.01 with Student’s *t* test, in comparison to W0 (**a**) or X0 (**b**). **º***p* < 0.05 with Student’s *t* test, in comparison to QsuB

To investigate any potential modification in stem mechanical properties, Young’s modulus was measured by the three-points bending method to evaluate the inflorescence stem stiffness of the engineered plants. Young’s modulus of pC4H::QsuB and W1 stems were similar to W0 while W2-4, W3-5, W4-8, and W4-1 had a significant reduction in stem stiffness, reflected by a decrease of Young’s modulus values (Fig. 7a).

As in W4 engineered lines, X4-10 and X4-12 stem stiffness was significantly reduced by about 30% compared to X0 (Fig. 7b). In conclusion, engineered lines overexpressing the galactan biosynthetic pathway in fiber tissues are affected in stem stiffness independently of the expression of *QsuB*. However, some lines had only minor reduction in stiffness, and none of the plants showed any morphological differences when compared to the control lines.

### Saccharification efficiency of engineered lines

**Fig. 8:**
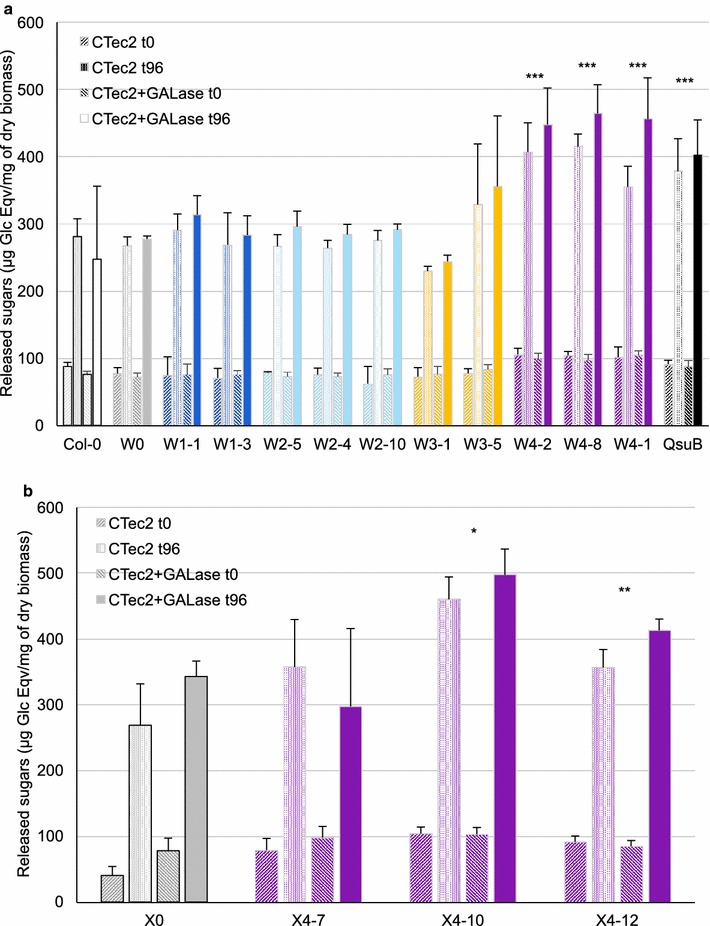
Saccharification assays of multiple traits engineered lines. Biomass from dry stems of W- (**a**) and X-(**b**) engineered lines after hot water pretreatment was assessed for saccharification efficiency with CTec2 enzyme cocktail alone or CTec2 complemented with endo-β-1,4-galactanase at 0 and 96 h. The amount of released sugars was tested with a DNS assay. Bars: SD, *n* = 3. The sugar release following enzyme treatment was analyzed by 2-factor ANOVA, followed by Dunnett’s multiple comparison test. *p < 0.05, **p < 0.01, ***p < 0.001 in comparison to W0 (**a**) or X0 (**b**). The effect of adding galactanase was significant across all the lines (ANOVA, *p* < 0.01) but the interaction between galactanase and genotype was not significant

Saccharification experiments were performed on engineered biomass samples pretreated with hot water at 120 °C, using the Cellic CTec2 commercial cellulase enzyme blend alone or in combination with an endo-β-1,4-galactanase. Saccharification efficiency was evaluated by measuring the amount of reducing sugars (pentoses and hexoses) released at different time points using a colorimetric DNS assay. Compared to W0 and Col-0 controls, biomass from W1, W2, and W3 lines released a similar amount of fermentable sugars, ranging between 260 and 290 µg Glc eqv/mg of dry biomass or slightly less in the case of the W3-1 line (Fig. 8). However, the W4 lines (W4-1, W4-2, and W4-8) released 30 to 54% more reducing sugars when hydrolyzed by cellulases. Compared to Cellic CTec2 hydrolyzed W0 stem biomass, QsuB and W4-8 stem biomass treated with the same enzymatic cocktail released 35 and 48% more reduced sugars, respectively. In both W- or X-genetic backgrounds, very high saccharification efficiencies were obtained with biomass from plants carrying the construct C4. In X4 plants, the efficiency of both hot water pretreatment and saccharification was highly increased. Additional treatment with an endo-β-1,4-galactanase had a significant effect (ANOVA, *p* < 0.01) and on the average released 9% additional sugars. In the W4 lines, 73% more sugar was released with the combination of Ctec2 and galactanase as compared to the control plants.

## Discussion

### Overexpression of *GALS1*, *UGE2*, *URGT1,* and *NST1* and additional expression of *QsuB* in W- and X-Arabidopsis genetic backgrounds

In this report, multiple traits beneficial to biofuel production were combined in Arabidopsis using the jStack cloning method. Up to three individual traits were simultaneously introduced via a gene stacking approaches into wild-type (W-) and xylan-engineered (X-) backgrounds (Figs. 1, 2). Plants were generated to overexpress three key genes (*GALS1*, *UGE2,* and *URGT1*) involved in galactan biosynthesis, and showed increased expression under both constitutive and fiber-specific promoters in T3 generation (Fig. 3). These W1 and W2 lines did not show a morphological phenotype compared to Col-0, and exhibited similar development during their life cycles. Lines expressing the C4 construct displayed opposite phenotypes in the W- and in X-genetic backgrounds (Fig. 2). While the inflorescence stems of engineered lines tended to be shorter in the W-background, they had the same height or were taller in the X-genetic background.

Lines in the W- or X-genetic backgrounds transformed with all the traits in combination (construct C5) show poor growth and weak stems. Thus, this may be indicative that brute-force overexpression strategies may have been exhausted and, in this specific combination, it is quite possible that we have reached the limits of genetic engineering. Alternatively, using different promoters or expressing the *NST1* APFL independently from *UGE2* could show different outcomes, as the increased expression of *NST1* results in higher than optimal level of expression of *QsuB* when the promoter (*pCesA7*) used to drive its expression is induced by the NST1 transcription factor. Testing different promoter combinations could likely overcome the detrimental phenotype.

Using the jStack cloning method, we generated engineered lines carrying all the transgenes at the same locus and the use of a tissue-specific promoter allowed the coordinating spatio-temporal expression of the desired transgenes. Because we had experience with a limited number of strong fiber-specific promoters, we decided to use the 2A peptide to stack genes in addition to the jStack approach. This technique has previously been used successfully by others and in our laboratory. However, in W3-1 and W3-5 stem sections, the presence of the APFL driven by NST1 did not appear to result in a substantial increase of cell wall thickness in fibers compared to what we had observed in previous studies [19, 24]. As mentioned before, a better comprehension of the network regulation of cell wall-related genes and a different sequence arrangement could possibly lead to the desired increase in fiber cell wall density. Alternatively, it is possible that the enhancement of galactan biosynthesis in fibers with promoters that are responsive to NST1 may cause a drastic substrate competition for UDP-glucose between the cellulose and galactan biosynthesis pathways. That could perhaps lead to reduced ability to also increase cellulose and hemicellulose biosynthesis by overexpression of NST1.

### Engineered plants showed an increase of galactan in fibers

Previously, we showed that the overexpression of *UGE2 and GALS1* driven by 35S promoter in Col-0 plant leads to 80% more galactose in the cell wall of stems [19]. Here, we engineered plants able to accumulate up to 150% more galactose in stem cell walls compared to the wild type. However, our qPCR data did not confirm increased *URGT1* expression in all of our engineered lines relative to the control plants (Fig. 3a, b). Some lines did show a three- to 11-fold increase in *URGT1* expression (i.e., W4-1 and X4-12) and these showed the highest increase in cell wall galactose accumulation, indicating the importance of boosting the expression of this gene. Expressing *URGT1* under a stronger fiber-specific promoter or inserting its native introns could perhaps result in producing even more galactan in cell walls due to increased transport of UDP-galactose into the Golgi lumen. LM5 immunodetection showed that the additional galactose is located in fibers and assembled into galactan polymers (Fig. 4). Moreover, by introducing more galactan in fibers of XE, we demonstrated that it is conceivable to produce dicot plants designed to have reduced xylan content and a high amount of C6 sugars in the secondary cell wall of fibers (Figs. 4, 5, Additional file 2). The C6/C5 sugar ratio determined by TFA hydrolysis of cell wall preparations of the X4-12 plant line is 2.66 as compared to 0.80 in Col-0, corresponding to a 3.3-fold increase (Additional file 2). Assuming that TFA hydrolysis-resistant cellulose levels are the same in all the plants, we estimated a C6/C5 ratio of 6.3 in the best X4 line versus 2.6 in Col-0, corresponding to a 2.4-fold increase.

We also demonstrated that the low lignin trait conferred by QsuB engineering is compatible with our hexose-enrichment strategies. Indeed, QsuB expression does not interfere with the accumulation of galactose (Fig. 5) in contrast to the biomass densification trait controlled by APFL. In lines W4-8 and W4-1, QsuB and galactan biosynthesis gene expression seems to have a synergistic effect (Fig. 5a).

### Galactan content in fibers can impact stem stiffness

Modifying the composition of cell walls in stem fibers could affect the mechanical properties of the entire organ. Such modifications could be advantageous to prevent lodging or, on the contrary, enhance lodging susceptibility. To address this question beyond the macroscopic phenotype, we tested stem stiffness with a three-point bending test. Our results showed no substantial effect on stem stiffness in most of the lines (Fig. 7). However, some lines carrying the C4 construct, in both the W- and X-genetic background, showed a decrease in stiffness, which was not observed in the QsuB control line. This decrease in stiffness was observed in the same lines where the elevated contents of galactose were found (Figs. 5, 7). In these plants, we may have reached the critical point where accumulation of galactan impacts tissue properties and consequently stem mechanical properties. Indeed, galactose content in the cell wall has been demonstrated to impact mechanical properties of Arabidopsis leaves [29]. However, we cannot conclude from the data whether the decreased stiffness in the W4 and X4 lines is due only to the high galactan content or due to the combination of high galactan with low lignin mediated by *QsuB* expression.

### Optimized C6/C5 sugar ratios are compatible with saccharification improvement traits

Because most microbes used for conversion into biofuels and bioproducts are more efficient in metabolizing C6 than C5 sugars, we aimed to increase the C6/C5 sugar ratio to optimize plant biomass for biofuels production.

The saccharification assays conducted in this study showed that our engineered plants released more C6 sugars in both the wild-type and in a low C5 sugar (XE) background (Fig. 8). The best engineered line resulted from fiber-specific overexpression of galactan biosynthesis-related genes and the bacterial gene *QsuB*. Previously, we have shown that the expression of *QsuB* itself doubled the saccharification efficiency of Arabidopsis biomass [12]. *QsuB* expression leads to a decrease of G/S ratio and an increase of H-units in lignin, and biomass of QsuB-expressing plants is more easily hydrolyzed by the Cellic CTec2 enzymatic cocktail than control biomass [12]. In our experiments, the same phenomenon was observed with the QsuB control line. In W4 and X4 lines, the improvement in saccharification due to QsuB combined with the galactan increase in fibers could have an additive effect, resulting in an even better sugar release.

## Conclusion

Here, we have engineered plants with up to a 3.3-fold increase of the C6/C5 sugar ratio in the TFA-hydrolyzable biomass fraction and with reduced lignin. No morphological differences were observed in these plants, except for a slight decrease in stem stiffness and change in height in some of the lines. No significant changes in stem biomass accumulation were observed. The approach demonstrated here can be transferred to bioenergy crops such as poplar (*Populus* sp.) and possibly also to bioenergy grasses in which galactan is not an abundant polymer. In the absence of mutants in xylan biosynthesis, an alternative method to obtain a similar reduction in xylan specifically in fibers could be achieved by targeted CRISPR/Cas9 mutagenesis directed against a xylan biosynthesis gene in fiber cells [30]. The effect of changing the biomass in bioenergy crops on agronomical performance is a key question to be resolved and ultimately will require field tests. The galactan-engineered lines described in this study are a valuable tool for further investigation of the potential relationship between galactan content in the secondary cell wall, mechanical properties, and stress responses.

## Methods

### jStack cloning

The jSTACK DNA assembly method was used as previously described to assemble the five binary plasmids designed for plant cell wall engineering (Fig. 1) [25]. Level 0, level 1, and level 2 assemblies are further detailed in Additional file 4. The binary vector pYB3301 conferring Basta^**®**^ (glufosinate) resistance to transformed plants was used for all gene stacks [25]. To ensure that all gene cassettes were correctly assembled, junctions were sequenced using the SimplySeq DNA Sequencing service provided by Quintara Biosciences (South San Francisco, CA). All sequences and plasmids developed in this project are further described in Additional files 3 and 4 and will be made publicly available through the Inventory of Composable Elements (ICE) repository [31].

### Plant lines and growth conditions

All Arabidopsis (*Arabidopsis thaliana* (L.) Heyhn.) wild-type and mutant plant lines used in this study are in the Columbia (Col-0) background. *pC4H::QsuB* and *irx7/irx7 pVND7:IRX7* lines have been described before [12, 22] and were obtained from the Joint BioEnergy Institute Registry (http://www.acs-registry.jbei.org). After being stratified at 4 °C for 4 days, seeds were grown in soil at 22 °C in a 10-h photoperiod for 4 weeks and then moved to a 16-h photoperiod. *Agrobacterium tumefaciens* strain GV3101 was transformed with the generated plasmids by electroporation [32] and used for plant transformation by floral dip [33]. Seeds from the transformed plants were harvested, sterilized in 10% bleach in ethanol for 10 min, and then grown on plates containing MS media (0.5 × Murashige and Skoog salts, 7 g/l agar, 10 g/l sucrose) with 25 μg/ml glufosinate ammonium for selection and stratified for 4 days at 4 °C. Plates were then transferred to a growth chamber at 22 °C with 10-h photoperiod for 7–10 days. Glufosinate-resistant seedlings were transferred to soil and grown for 4 weeks at 22 °C in 10-h photoperiod and then transferred to a 16-h photoperiod.

### Transgene expression analysis by qPCR

Samples of seven-week-old main inflorescence stems were ground in liquid nitrogen and RNA was extracted using TRIzol reagent (Thermo Fisher). RNA was treated with DNase I (AMPD1-1KT, Sigma-Aldrich) to eliminate DNA contaminants and cDNA was synthesized using iScript Reverse Transcription Supermix (Bio-Rad, 1708840) with 750 ng RNA as a template in a 10 μl reaction volume. The expression level of *GALS1*, *UGE2*, *URGT1*, *NST1,* and *QsuB* was measured by quantitative PCR using the StepOnePlus Real-Time PCR system (Applied Biosystems) according to the conditions described in Czechowski et al. [34] using StepOne 2.0 software (Applied Biosystems). Primers were designed to amplify cDNAs from both endogenous and transgenic mRNAs of *GALS1*, *UGE2*, *URGT1,* and *NST1*. The expression level measured for these genes is a combination of the expression levels of the endogenous copy and the transgenic copy. Gene expression was analyzed using the *ΔΔC*_*T*_ method [35] and normalization against the geometric mean of the transcript levels of three reference genes [36]. The constitutively expressed reference genes used (*UBQ10* (At4g05320), *PP2AA3* (At1g13320), *MON1* (At2g28390)) have been validated by Czechowski et al. [34]. Primer sequences are available in Additional file 3.

### Immunofluorescence microscopy

The top and base 2.5 cm of main stems from seven-week-old plants (three stems/line) were harvested and fixed overnight at 4 °C in fixative solution (4% paraformaldehyde in 50 mM piperazine-N–N′-bis(2-ethanesulphonic acid), 5 mM EGTA, pH 6.9). Fixed stem sections were embedded in 7% agarose and 100 µm thick sections were generated using a Leica VT1000S vibratome. Stem sections were labeled with monoclonal antibody LM5 (PlantProbes, Leeds, UK), which recognizes 1,4-linked β-galactan [37, 38]. Stem sections were washed three times with phosphate-buffered saline (PBS) and incubated for 1.5 h at room temperature with LM5 or LM10 antibody diluted tenfold in PBS with 5% milk protein. After three washes in PBS, stem sections were incubated for 1.5 h in the dark at room temperature with anti-rat IgG secondary antibody coupled with FITC (Fluorescein IsoThioCyanate) diluted 100-fold in PBS with 5% milk protein. Stem sections were finally washed three times with PBS and stored overnight at 4 °C in the dark. Immunostained stem cuts were mounted on slides in a glycerol anti-fade solution (CitiFluor AF1, Agar Scientific) and pictures were taken using a DM6 B epifluorescence microscope (Leica) under blue light (L5 filter, Leica). Images were acquired with a C11440 Hamamatsu camera monitored by the LAS X software (Leica) and then analyzed with ImageJ [39].

### Monosaccharide composition analysis

Whole inflorescence stems from dry mature plants were ground in vials with five metal beads using a Retch mill, and alcohol-insoluble residue (AIR) was prepared and enzymatically destarched as described by Harholt et al. [40]. AIR samples (1 mg) were subsequently hydrolyzed with 2 M trifluoroacetic acid (TFA) for 1 h at 120 °C. TFA was removed under vacuum overnight and hydrolysis products suspended in 1 ml water at 30 °C for 30 min. The monosaccharide composition was determined in 15-fold diluted hydrolysates by high-performance anion exchange chromatography coupled with a pulsed amperometric detection (HPAEC-PAD) using an ICS-5000 ion chromatography system with a CarboPac PA20 column (Dionex Thermo Scientific) as described [41, 42].

### Young’s modulus measurement

Sections of main inflorescence stems from 25 to 75 mm above the rosette were taken from seven-week-old plants and three-point flexural tests were performed using a 4500 series Instron universal testing machine (series IX automated materials testing system, http://www.instron.co.uk). Flexural three-point bending stiffness (Young’s modulus) was calculated according to the standard equations [43].

### Analysis of biomass saccharification efficiency

Saccharification efficiency was analyzed after hot water pretreatment of ground biomass from senesced stems. Samples (10 mg) were mixed with 340 μl of water and incubated for 30 min at 30 °C with shaking, autoclaved for 1 h at 120 °C, and cooled down to room temperature. Enzymatic saccharification was initiated by adding 650 μl of saccharification mix (75 mM citrate buffer pH 5; 115 μg/ml tetracycline; 1% v/w Cellic CTec2 enzyme mix (Novozymes)) to pretreated samples. In some samples, endo-1,4-β-galactanase from *Aspergillus niger* (Megazyme) was included at a concentration of 52 U/mg of biomass. The reaction was carried out at 50 °C for 96 h with shaking. Reducing sugar concentration was measured at *t* = 0 h, *t* = 20 h, *t* = 48 h, *t* = 72 h and *t* = 96 h using the colorimetric dinitrosalicylic acid (DNS) assay [44]. Samples were centrifuged for 3 min at 13,000x*g* to pellet biomass and a 10 μl aliquot of supernatant was diluted in 20 μl of 100 mM citrate buffer pH 5. Ninety microlitre of DNS reagent (0.4 M NaOH; 300 g/l KNa tartrate; 10 g/l DNS) was added and the mix was heated at 90 °C for 10 min. The absorbance was measured at 540 nm and reducing sugar concentration was quantified (in glucose equivalents) using glucose solutions as standards.

### Statistical analysis

Statistical analysis was done with Students *t* test or with ANOVA as indicated in the figure legends. ANOVA and multiple comparisons (by Tukey’s and Dunnett’s tests) were done using XLSTAT (Addinsoft, New York).

### Sequence IDs

The promoters and coding sequences used in the gene constructs relate to the following IDs: AtIrx5, At5g44030; AtIrx8, At5g54690; CesA7, At5g17420; GalS1, At2g33570; UGE2, At4g23920; URGT1, At1g76670; NST1, At2g46770; UBQ10, At4g05320; PP2AA3, At1g13320; MON1, At2g28390; QsuB, YP_001137362.1. For further details, see Additional file 3.

## Additional files

**Additional file 1.** Phenotypes of W5 and X5 engineered lines.

**Additional file 2.** Monosaccharide composition of stem cell walls of engineered lines. Alcohol-insoluble residue (AIR) of stem cell walls of W- (A) and X- (B) engineered lines, hydrolyzed with trifluoroacetic acid, and analyzed by HPAEC-PAD. Bars: SD, n = 3.

**Additional file 3.** Primers used for jStack cloning and qPCR transcript level monitoring.

**Additional file 4.** Summary of jStack constructs.
