# Supplementary material for: Gene stacking of multiple traits for high yield of fermentable sugars in plant biomass
Source: Biotechnol Biofuels. 2018 Jan 9;11:2. doi: 10.1186/s13068-017-1007-6 (PMC5759196; doi:10.1186/s13068-017-1007-6)
Supplement: Supplementary file 1 — Additional file 1. Phenotypes of W5 and X5 engineered lines. [file 13068_2017_1007_MOESM1_ESM.pdf]

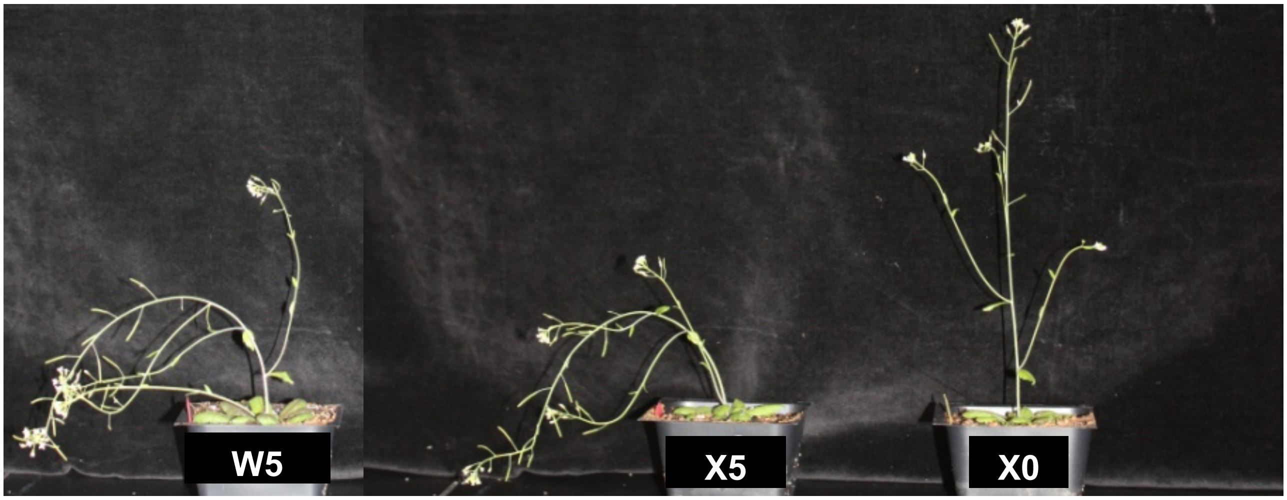

Additional file 1: Phenotypes of W5 and X5 engineered lines. . Pictures of engineered plants carrying the construct C5 in Col-0 wild type (W5) and *irx7/irx7* pVND7:IRX7 xylan-engineered (X5) backgrounds.
