# Supplementary material for: Gene stacking of multiple traits for high yield of fermentable sugars in plant biomass
Source: Biotechnol Biofuels. 2018 Jan 9;11:2. doi: 10.1186/s13068-017-1007-6 (PMC5759196; doi:10.1186/s13068-017-1007-6)
Supplement: Supplementary file 2 — Additional file 2. Monosaccharide composition of stem cell walls of engineered lines. Alcohol-insoluble residue (AIR) of stem cell walls of W- (A) and X- (B) engineered lines, hydrolyzed with trifluoroacetic acid, and analyzed by HPAEC-PAD. Bars: SD, n = 3. [file 13068_2017_1007_MOESM2_ESM.pdf]

A.

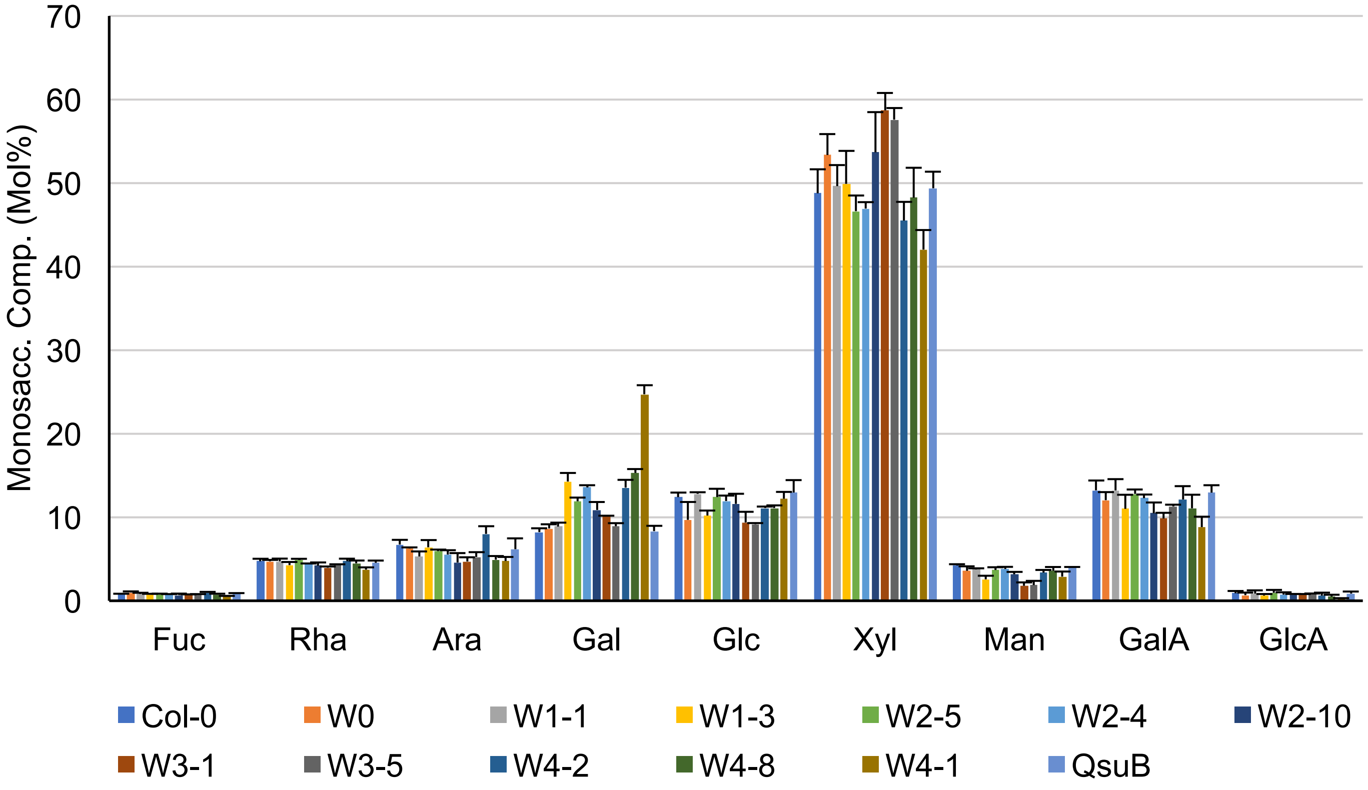

B.

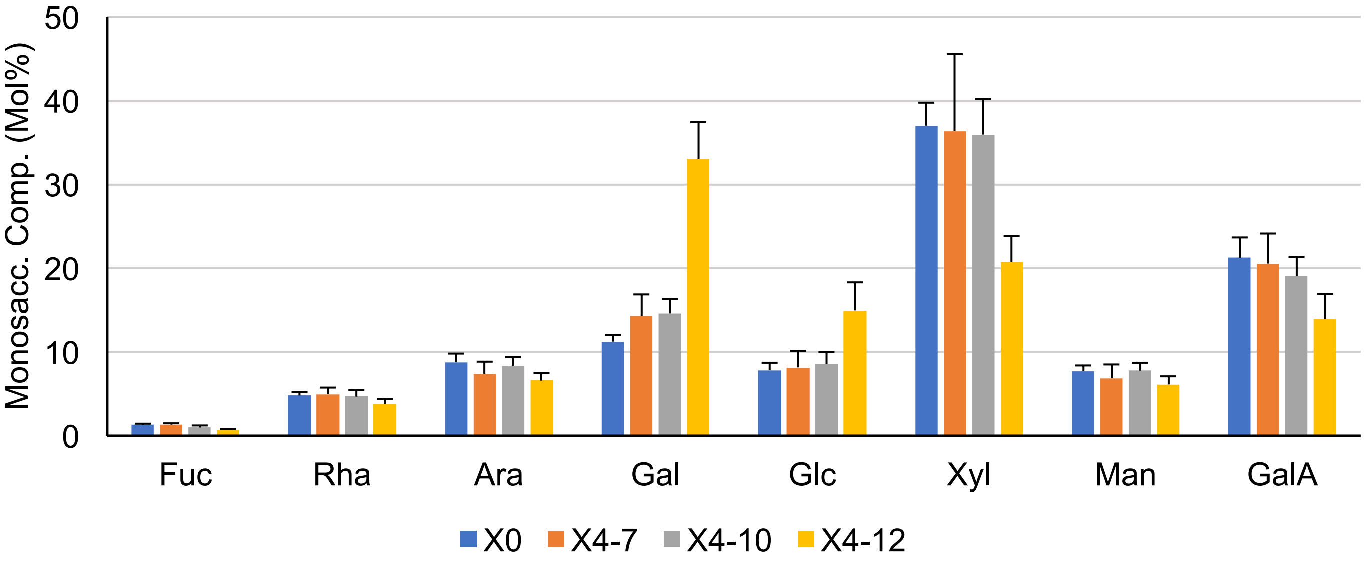

Additional file 2: Monosaccharides content in stem cell walls of engineered lines. Alcohol insoluble residue (AIR) of stem cell wall of W- (A) and X- (B) engineered lines, hydrolyzed with trifluoroacetic acid, and analyzed by HPAEC-PAD liquid chromatography. Bars: SD. n=3
