# Supplementary material for: Gene stacking of multiple traits for high yield of fermentable sugars in plant biomass
Source: Biotechnol Biofuels. 2018 Jan 9;11:2. doi: 10.1186/s13068-017-1007-6 (PMC5759196; doi:10.1186/s13068-017-1007-6)
Supplement: Supplementary file 4 — Additional file 4. Summary of jStack constructs. [file 13068_2017_1007_MOESM4_ESM.pdf]

construct name	Level	Description
**C1**	**Level 2**	**{L_tOcs}{P_nos}{C_GALS1}{T_tG7}{P_MAS}{C_UGE2}{T_AtAct2}{P_AtAct2}{C_URGT1}{T_tNos}**
pms6132	Level 1	{L_tOcs}{P_nos}{C_GALS1}{T_tG7}
pms6133	Level 1	{L_tG7}{P_MAS}{C_UGE2}{T_AtAct2}
pms6134	Level 1	{L_AtAct2}{P_AtAct2}{C_URGT1}{T_tNos}
**C2**	**Level 2**	**{L_tOcs}{P_AtC4H}{C_GALS1}{T_tG7}{P_AtIRX5}{C_UGE2}{T_AtAct2}{P_AtIRX8}{C_URGT1}{T_tNos}**
pms6135	Level 1	{L_tOcs}{P_AtC4H}{C_GALS1}{T_tG7}
pms6136	Level 1	{L_tG7}{P_AtIRX5}{C_UGE2}{T_AtAct2}
pms6137	Level 1	{L_AtAct2}{P_AtIRX8}{C_URGT1}{T_tNos}
**C3**	**Level 2**	**{L_tOcs}{P_AtC4H}{C_GALS1}{T_tG7}{P_AtIRX5}{C_NST-2A-UGE2}{T_AtAct2}{P_AtIRX8}{C_URGT1}{T_tNos}**
pms6135	Level 1	{L_tOcs}{P_AtC4H}{C_GALS1}{T_tG7}
pms6138	Level 1	{L_tG7}{P_AtIRX5}{C_NST-2A-UGE2}{T_AtAct2}
pms6137	Level 1	{L_AtAct2}{P_AtIRX8}{C_URGT1}{T_tNos}
**C4**	**Level 2**	**{L_tOcs}{P_AtC4H}{C_GALS1}{T_tG7}{P_AtIRX5}{C_UGE2}{T_AtAct2}{P_AtIRX8}{C_URGT1}{T_MAS}{P_ArCesA7}{C_QsuB}{T_tNos}**
pms6135	Level 1	{L_tOcs}{P_AtC4H}{C_GALS1}{T_tG7}
pms6136	Level 1	{L_tG7}{P_AtIRX5}{C_UGE2}{T_AtAct2}
pms6139	Level 1	{L_AtAct2}{P_AtIRX8}{C_URGT1}{T_MAS}
pms6140	Level 1	{L_MAS}{P_ArCesA7}{C_QsuB}{T_tNos}
**C5**	**Level 2**	**{L_tOcs}{P_AtC4H}{C_GALS1}{T_tG7}{P_AtIRX5}{C_NST-2A-UGE2}{T_AtAct2}{P_AtIRX8}{C_URGT1}{T_MAS}{P_ArCesA7}{C_QsuB}{T_tNos}**
pms6135	Level 1	{L_tOcs}{P_AtC4H}{C_GALS1}{T_tG7}
pms6138	Level 1	{L_tG7}{P_AtIRX5}{C_NST-2A-UGE2}{T_AtAct2}
pms6139	Level 1	{L_AtAct2}{P_AtIRX8}{C_URGT1}{T_MAS}
pms6140	Level 1	{L_MAS}{P_ArCesA7}{C_QsuB}{T_tNos}

Additional file 4: Summary of jStack constructs.
